# Ketoconazole Inhibits the Cellular Uptake of Anandamide via Inhibition of FAAH at Pharmacologically Relevant Concentrations

**DOI:** 10.1371/journal.pone.0087542

**Published:** 2014-01-23

**Authors:** Emmelie Björklund, Therése N. L. Larsson, Stig O. P. Jacobsson, Christopher J. Fowler

**Affiliations:** Department of Pharmacology and Clinical Neuroscience, Umeå University, Umeå, Sweden; University of Padova, Italy

## Abstract

**Background:**

The antifungal compound ketoconazole has, in addition to its ability to interfere with fungal ergosterol synthesis, effects upon other enzymes including human CYP3A4, CYP17, lipoxygenase and thromboxane synthetase. In the present study, we have investigated whether ketoconazole affects the cellular uptake and hydrolysis of the endogenous cannabinoid receptor ligand anandamide (AEA).

**Methodology/Principal Findings:**

The effects of ketoconazole upon endocannabinoid uptake were investigated using HepG2, CaCo2, PC-3 and C6 cell lines. Fatty acid amide hydrolase (FAAH) activity was measured in HepG2 cell lysates and in intact C6 cells. Ketoconazole inhibited the uptake of AEA by HepG2 cells and CaCo2 cells with IC_50_ values of 17 and 18 µM, respectively. In contrast, it had modest effects upon AEA uptake in PC-3 cells, which have a low expression of FAAH. In cell-free HepG2 lysates, ketoconazole inhibited FAAH activity with an IC_50_ value (for the inhibitable component) of 34 µM.

**Conclusions/Significance:**

The present study indicates that ketoconazole can inhibit the cellular uptake of AEA at pharmacologically relevant concentrations, primarily due to its effects upon FAAH. Ketoconazole may be useful as a template for the design of dual-action FAAH/CYP17 inhibitors as a novel strategy for the treatment of prostate cancer.

## Introduction

The endocannabinoid system, comprising the cannabinoid CB receptors, their endogenous ligands arachidonoylethanolamide (anandamide, AEA) and 2-arachidonoylglycerol (2-AG), and their synthetic and metabolic enzymes, are involved in a variety of regulatory pathways including the control of pain, appetite, reproduction, bone turnover and control of cancer [1]–[5]. AEA is removed from the extracellular space by a process of cellular uptake followed by enzymatic metabolism, primarily to arachidonic acid via the hydrolytic enzyme fatty acid amide hydrolase. The mechanism(s) whereby AEA crosses the plasma membrane are a matter of controversy [6], but once within the cell, a variety of carrier proteins (fatty acid binding proteins, heat shock protein 70 and albumin) transport this lipid either to sequestration sites, to intracellularly located binding sites on receptors, and/or to the catabolic enzymes [7], [8]. A fatty acid amide hydrolase [FAAH]-like transporter protein has also been suggested to act as an AEA transporter protein [9] but this has been disputed recently in this journal [10].

The main catabolic enzyme for AEA is the enzyme FAAH, which hydrolyses the endocannabinoid to give arachidonic acid and ethanolamine [11]. In addition to FAAH, AEA can act as a substrate for other enzymes, including cyclooxygenase-2 and lipoxygenases [12], and evidence is accruing to suggest that such pathways may have important pathophysiological relevance [13], [14]. AEA is also metabolised by several members of the CYP enzyme family including CYP3A4 and CYP4F2. CYP3A4 in human liver microsomes, for example, is responsible for the production of a family of epoxyeicosatrienoic acid ethanolamides, and the 5,6- derivative is a potent agonist at cannabinoid-2 receptors [15], [16].

Ketoconazole is a member of the azole family of antifungal reagents which exert their therapeutic effects by blocking fungal ergosterol synthesis via inhibition of sterol 14α-demethylase (CYP51) [17]. However, ketoconazole has additional effects upon other enzymes, including several human CYP isoforms (primarily CYP3A4, but also CYP17 involved in steroid biosynthesis [18], [19]) and upon the activity of 5-lipoxygenase [20]. Given that AEA interacts with CYP3A4 and lipoxygenases, there is an overlap between the targets for this endocannabinoid and for ketoconazole, raising the possibility that ketoconazole may interact directly with the endocannabinoid system. In the present study, we demonstrate that ketoconazole can affect AEA uptake and hydrolysis at pharmacologically relevant concentrations.

## Methods

### Compounds

Ketoconazole, sulfaphenazole, quinidine and nefazodone were obtained from Sigma-Aldrich Inc, St. Louis, (MO, U.S.A.). Non-radioactive AEA, URB597 (cyclohexylcarbamic acid 3′-carbamoylbiphenyl-3-yl ester) and JZL184 (4-nitrophenyl-4-(dibenzo[d][1], [3]dioxol-5-yl(hydroxy)methyl)piperidine-1-carboxylate) were obtained from Cayman Chemical Co., Ann Arbor, (MI, U.S.A.). AM404 (*N*-(4-hydroxyphenyl)arachidonylamide) and OMDM-2 ((9Z)-*N*-[1-((R)-4-Hydroxbenzyl)-2-hydroxyethyl]-9-octadecenamide) were obtained from Tocris Bioscience, Ellisville, (MO, U.S.A.). [Arachidonyl 5,6,8,9,11,12,14,15-^3^H]AEA (for uptake experiments, specific activity 7.4 TBq mmol^−1^), [ethanolamine-1-^3^H]AEA (for FAAH experiments, specific activity 2.2 TBq mmol^−1^), [palmitoyl 9,10-^3^H] palmitoylethanolamide ([^3^H]PEA, specific activity 2.2 TBq mmol^−1^), [arachidonoyl 5,6,8,9,11,12,14,15-^3^H]2-AG (for uptake experiments, specific activity 7.4 TBq mmol^−1^) and [glycerol-1,2,3-^3^H]2-AG (for hydrolysis experiments, specific activity 1.48 TBq mmol^−1^) were obtained from American Radiolabeled Chemicals Inc., St Louis, MO, U.S.A.

### Cell cultures

All cells were grown in 75 cm^2^ flasks at 37°C with 5% CO_2_ in humidified atmospheric pressure. Cells were spilt into new flasks once or twice per week. All culture media were supplemented with 1 IU mL^−1^ penicillin, 1 µg mL^−1^ streptomycin and 10% foetal bovine serum. HepG2 human liver hepatocellular carcinoma cells (passage 104–124) were obtained from Health Protection Agency Culture Collections (Porton Down, U.K.) and grown in William's E Medium Glutamax. CaCo-2 human epithelial colorectal adenocarcinoma cells (passage 31–97), originating from the American Type Culture Collection (Manassas, VA, U.S.A.) were cultured in Dublecco's Modified Eagle Medium (DMEM), 1% non essential amino acids (NEAA), and 2 mM L-glutamine. SH-SY5Y human neuroblastoma cells (passage 28) obtained from European Collection of Cell Cultures (ECACC; Porton Down, UK) were cultured in MEM with Earl's salts, 1% NEAA, 1% L-glutamine. Rat C6 glioma cells (passage range 18–22) obtained from ECACC were cultured in Ham's F-10 medium with 2 mM glutamine. PC-3 human prostate cancer cells (passage range 18–20) were obtained from DSMZ (Braunschweig. Germany) and cultured in Ham's F-10 medium, 2 mM L-glutamine, 10% foetal bovine serum and penicillin + streptomycin. Media and all supplements were obtained from Invitrogen Life technologies (Stockholm, Sweden).

### Uptake assays

The procedure was initially developed by Rakhshan *et al.* [21] and modified by Sandberg and Fowler [22]. On the day before the experiments, cells were plated in transparent 24-well plates at a density of 2×10^5^ cell per well in 400 µL of medium. After incubation over night in an incubator at 37°C and 5% CO_2_, cells were washed twice with 400 µL warm KRH-buffer (Krebs-Ringer HEPES buffer pH 7.4 in MilliQ-water; 120 mM NaCl, 4.7 mM KCl, 2.2 mM CaCl_2_, 10 mM HEPES, 1.2 mM KH_2_PO_4_, 1.2 mM MgSO_4_) with 1% BSA first, then in the same manner without BSA. KRH-buffer (340 µL) with 0.1% fatty acid free BSA was added together with 10 µl of test compounds (ketoconazole, sulfaphenazole, quinidine, nefazodone or URB597 (cyclohexylcarbamic acid 3′-carbamoylbiphenyl-3-yl ester)) or vehicle. The concentration of solvent (DMSO or EtOH) was kept constant at ≤1% in all assays, also in the blanks. After 10 minutes of preincubation at 37°C, 50 µl of the tritiated substrate ([^3^H]AEA, labelled in the arachidonate part of the molecule, [^3^H]PEA or [^3^H]2-AG) at a final concentration of 100 nM in KRH-buffer was added and the cells were incubated for 4 minutes at 37°C, unless otherwise shown. To stop the reaction, plates were placed on ice and washed three times with 500 µl KRH-buffer containing 1% BSA. Finally 500 µl 0.2 M NaOH was added and plates were incubated at 75°C for 15 minutes to solubilise cells. Following cooling to room temperature, 300 µl from each well was transferred to a scintillation vial and 4 ml of scintillation liquid was added. The tritium content was then measured by scintillation spectroscopy with quench correction. In a typical experiment where ∼30000 d.p.m. of ligand is added to the wells, the total recovery of tritium after an incubation time of 5 min is roughly 1000–2000 d.p.m. for experiments with cells (depending of course upon the cell type used), whilst the recovery is in the region of 100 d.p.m. is found for wells alone.

### FAAH activity measurements

For the cell-free assays, cells were washed twice with 10 ml of cold PBS (phosphate-buffered saline), followed by the addition of 5 ml PBS and the flasks were placed on ice. The cells were detached by using a rubber policeman and collected to a 15 ml tube, and thereafter 5 ml PBS was used to rinse the flask. The cells were centrifuged for 5 min at 200 g at 4°C, and the pellet was resuspended in 1 ml 10 mM Tris-HCl buffer, pH 9. Aliquots were stored at −80°C until assayed for FAAH activity. On the day of experiment, the aliquots were thawed and the protein content was measured with bovine serum albumin used as standard [23]. The FAAH activity assay was performed essentially as described by Boldrup *et al.* [24]. Briefly, 175 µl of the protein solution containing 2.5 µg protein in 10 mM Tris-HCl buffer, pH 9, was added to each test tube. [^3^H]AEA (substrate labelled in the ethanolamine part of the molecule, 25 µL, final concentration 0.5 µM, in a buffer containing 1% w v^−1^ fatty acid free BSA and 10 mM Tris-HCl buffer pH 9) was added and incubated for 10 min in 37°C. Reactions were stopped by adding 80 µl of charcoal in 320 µl of 0.5 M HCl followed by vortex mixing, and the test tubes were placed on ice for 5 minutes, and then adjusted to room temperature for 30 min before centrifugation for 10 min. Aliquots (200 µl) of the supernatant were transferred into scintillation vials and 4 ml of scintillation liquid were added. The tritium content was then measured by scintillation spectroscopy with quench correction. Blank values were obtained using buffer in place of cell lysate.

For the FAAH assays in intact C6 glioma cells, the initial part of the assay was essentially the same as for the uptake assays, albeit with [^3^H]AEA labelled in the ethanolamine rather than the arachidonate part of the molecule. Following incubation for 10 min at 37°C, reactions were stopped by addition of 120 µl of charcoal in 480 µl of 0.5 M HCl and aliquots (600 µl) transferred to test tubes. The samples were then centrifuged and aliquots (200 µl) of the supernatant were collected and analysed as described above. Blank values were obtained in the absence of cells. The same assay was used for 2-AG hydrolysis, using [^3^H]2-AG labelled in the glycerol rather than the arachidonate part of the molecule.

### Statistical analyses

One-way ANOVAs, pI_50_ and IC_50_ values were determined using the GraphPad Prism computer programme (GraphPad Software Inc., San Diego, CA, USA). The pI_50_, and thereby IC_50_ values, were calculated using the built-in equation ‘log (inhibitor) vs. response – variable slope (four parameters)’ from the data expressed as % of vehicle controls using top (i.e. uninhibited) values of 100% and bottom (residual activity) values that were either set to zero or allowed to float. In cases where the residual activity was >0, the two models were compared using Akaike's Informative Criteria, to determine which model was the most likely.

## Results

### Effect of ketoconazole upon the uptake of [^3^H]AEA by HepG2, CaCo2 and PC-3 cells

**Figure 1:**
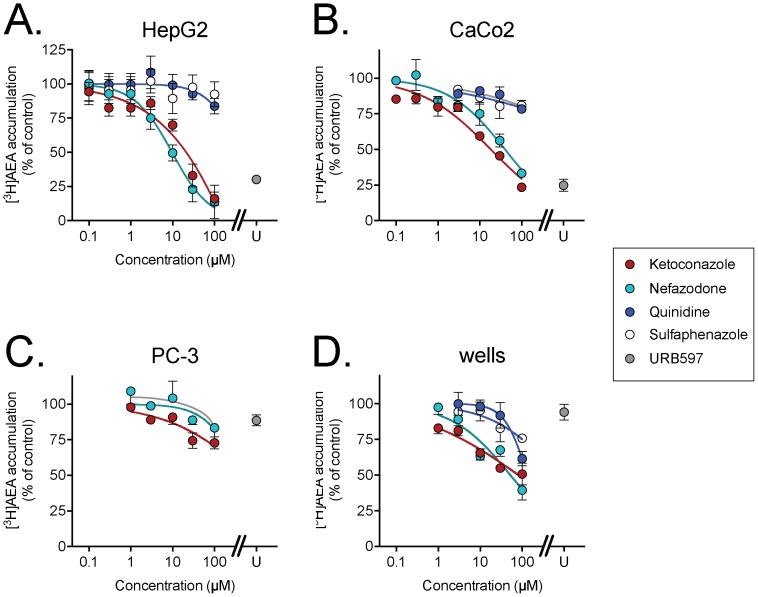
Inhibition of [^3^H]AEA uptake by ketoconazole, nefazodone, quinidine and sulphenazole. The panels show the data for A, HepG2 cells; B, CaCo2 cells; C, PC-3 cells and D, adsorption to wells alone. The cells (or wells) were preincubated with the compounds for 10 min prior to addition of [^3^H]AEA (assay concentration 100 nM) and incubation for a further 4 min. For comparative purposes, the effect of the selective FAAH inhibitor URB597 (“U”, 1 µM) is indicated. Shown are means ± s.e.m. (when not enclosed by the symbols), n = 3–9 except for 100 µM nefazodone in the HepG2 cells, where n = 2.

**Figure 2:**
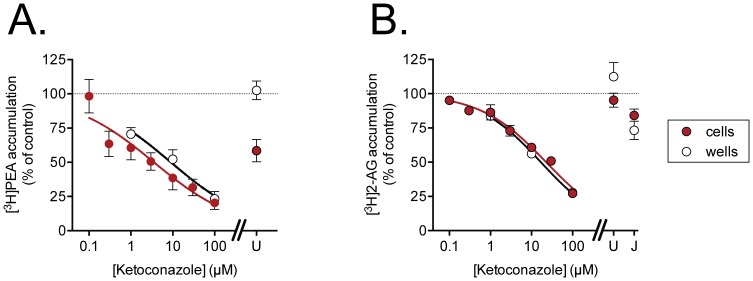
Inhibition of A. [^3^H]PEA and B. [^3^H]2-AG uptake by ketoconazole. The panels show the data for the uptake into HepG2 cells (filled symbols) or adsorbtion by wells (open symbols). The cells (or wells) were preincubated with ketoconazole for 10 min prior to addition of [^3^H]PEA or [^3^H]2-AG as appropriate (assay concentration 100 nM) and incubation for a further 4 min. For comparative purposes, the effects of the selective FAAH inhibitor URB597 (“U”, 1 µM) and (for 2-AG) the selective MGL inhibitor JZL184 (“J”, 100 µM) are indicated. Shown are means ± s.e.m. (when not enclosed by the symbols), n = 3.

The ability of ketoconazole to affect the uptake of [^3^H]AEA by HepG2 and CaCo-2 cells is shown in Fig. 1A and B. The compound concentration-dependently inhibited uptake by both cells with pI_50_ values (with IC_50_ values in parentheses) of 4.77±0.09 (17 µM) and 4.73±0.08 (18 µM) for HepG2 and Caco2 cells, respectively. Ketoconazole was also found to inhibit the uptake of the related non-cannabinoid lipid [^3^H]PEA and of [^3^H]2-AG by HepG2 cells, with pI_50_ values of 5.45±0.16 (IC_50_ value 3.5 µM) and 4.64±0.06 (IC_50_ value 23 µM), respectively (Fig. 2A and B). AEA binds avidly to wells, although the level of binding is very low relative to the levels of cellular uptake under the assay conditions used here. In the present study, for example, the tritium recovered after incubation of AEA with the CaCo2 cells for 4 min was ∼12-fold higher than for the wells alone. However, the binding of AEA to the wells has been found to be inhibited by uptake inhibitors such as AM404 [25] and OMDM-2 [26] at concentrations similar to those needed to block cellular uptake [27], [28]. The same was found to be true for ketoconazole, which inhibited the binding of AEA to wells with a pI_50_ value of 4.09±0.19 (IC_50_ value 82 µM) (Fig. 1). The compound also inhibited the binding of [^3^H]PEA and [^3^H]2-AG to the wells over the same concentration range (Fig. 2).

**Figure 3:**
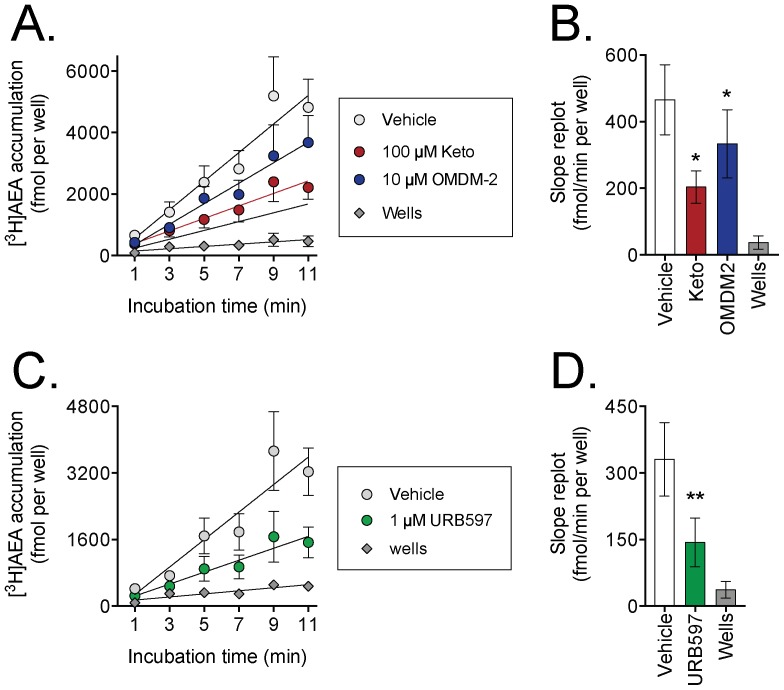
Time course of the effects of ketoconazole and URB597 upon the accumulation of [^3^H]AEA by HepG2 cells. In Panels A and C, the individual values at each time point are shown (means ± s.e.m., unless enclosed by the symbols, n = 6). For both Panels, two-way ANOVA with repeated time measures for the data with the cells gave a significant (P<0.05) interaction time x treatment. Regression lines for each experiment were determined and the slopes, i.e. the rates of uptake are shown in Panels B and D as means ± s.e.m., n = 6. The vehicles used were A, ethanol; B, DMSO. *P<0.05, **P<0.01 *vs*. vehicle, either using Dunnett's multiple comparisons test following significant one-way repeated ANOVA not assuming sphericity for the three conditions with cells (Panel C) or for a two-tailed paired t-test for the two conditions with cells (Panel D).

The interaction of AEA with the culture wells and its inhibition by ketoconazole is a potential confounding factor in the interpretation of the data. In the uptake experiments, subtraction of well data from the cell data is not appropriate - the cells cover the wells and thus reduce the available well surface area for binding, see [29]. As pointed out above, the binding to wells in absolute terms is low compared with the cellular uptake under the conditions used here. Nonetheless, the ability of the compounds to reduce the retention of ligand by the wells is an issue because it indicates that the compound can affect non-specific associations of the endocannabinoids, and may do the same in cells. One way of circumventing this issue is to investigate the time-dependency of the uptake. The rapid and reversible non-specific association of AEA with cell plasma membranes and with wells will not change over time, since there is little depletion of the ligand in the medium. In contrast, the accumulation of label over time will reflect the true cellular uptake. We have previously shown in P19 mouse embryonic carcinoma cells that there is a linear relationship between the amount of [^3^H]AEA retained by the cells and the incubation time, measured between 45 s and 15 min. The slope of the lines reflect the rate of cellular uptake, and this was found to be saturable with respect to the AEA concentration (K_m_ value 1 µM) [22]. A similar result was found for ND7/23 mouse neuroblastoma x rat dorsal root ganglion neurone hybrid cells [30]. In contrast, the extrapolated initial association of AEA with the cells at t = 0 showed no saturability [22], [30]. Thus, by following the accumulation of [^3^H]AEA over time and measuring the slope, effects of a compound upon the cellular uptake of AEA can be separated from its non-specific effects upon the association of the endocannabinoid with the available surface, be it cells or wells. Using this approach, we found that ketoconazole significantly reduced the rate of cellular accumulation of [^3^H]AEA by the HepG2 cells, as did the FAAH inhibitor URB597 [31] and OMDM-2 (Fig. 3A,B). For the ten experiments where slopes were determined (the six shown in Fig. 3, and four undertaken for ketoconazole and URB597, but not OMDM-2, using time points of 1, 4, 7 and 10 min), the slopes seen with ketoconazole, OMDM-2 and URB597 were 37% (29–46%), 68% (42–95%) and 36% (27–46%) (means and 95% confidence interval) of the corresponding vehicle controls, respectively. Under these conditions, the rate of increase of retention of [^3^H]AEA by the wells alone over time was extremely low. These data indicate that ketoconazole can produce both a non-specific effect upon the retention of [^3^H]AEA but also an inhibition of the cellular uptake of the ligand.

**Figure 4:**
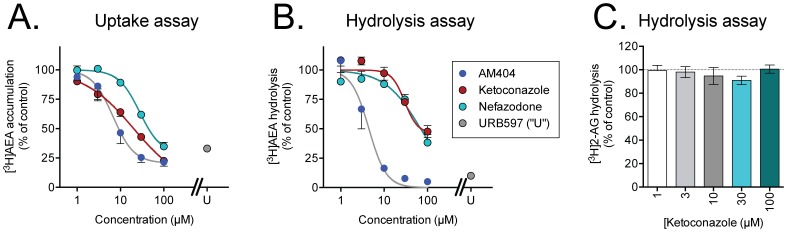
Effects of ketoconazole, nefazodone, AM404 and URB597 (“U”, 1 µM) upon A, the uptake of [^3^H]AEA; B, the hydrolysis of [^3^H]AEA and C, the hydrolysis of [^3^H]2-AG by C6 glioma cells. The compounds were preincubated with the cells for 10-AG (100 nM final concentration) and incubation for a further 10 min at 37°C. For the uptake experiments, the tritium label was on the arachidonoyl side chain, whilst for the hydrolysis experiments, the label was on the ethanolamine group (AEA) or the glycerol group (2-AG). Shown are means ± s.e.m. (when not enclosed by the symbols), n = 3, except for ketoconazole in Panel B, where n = 4.

The above conclusion was reinforced by experiments in C6 rat glioma cells, where the effects of ketoconazole, and for comparative purposes the prototypical endocannabinoid reuptake inhibitor AM404 upon the uptake of AEA were compared with a functional measure of intracellular accumulation, namely the production of [^3^H]ethanolamine from the intracellularly FAAH-catalysed hydrolysis of [^3^H]AEA labelled in this part of the molecule. C6 glioma cells have a high expression of FAAH and are thus a useful system to study the hydrolysis of internalised AEA. Both compounds inhibited the production of [^3^H]ethanolamine over a broadly similar concentration range as required for inhibition of the uptake of [^3^H]AEA (labelled in the arachidonoyl part of the molecule) (Fig. 4A and B). The pI_50_ values (with IC_50_ values in parentheses) for inhibition of uptake and hydrolysis, respectively, were: ketoconazole, 4.69±0.04 (20 µM) and 4.53±0.07 (29 µM; based on an inhibitable fraction of 54±7%); AM404, 4.93±0.09 (12 µM) and 5.35±0.07 (4.5 µM). In contrast, ketoconazole did not affect the hydrolysis of 2-AG over the concentration range tested (Fig. 4C).

In order to determine whether other compounds able to interact with CYP enzymes also affect [^3^H]AEA uptake, the CYP3A inhibitor nefazadone, the CYP2D6 inhibitor quinidine and the CYP2C9 inhibitor sulfaphenazole were investigated. Quinidine and sulfaphenazole had at best minor effects upon the cellular accumulation of AEA into HepG2 and CaCo2 cells. However, robust inhibition of the uptake of AEA into both HepG2 and Caco2 cells and of the association of AEA by the wells was seen with nefazodone (Fig. 1). The pI_50_ values (with IC_50_ values in parentheses) for nefazodone were: HepG2 cells, 5.02±0.09 (9.7 µM); CaCo2 cells, 4.40±0.10 (40 µM); wells, 4.28±0.11 (52 µM). As with ketoconazole, nefazodone inhibited the production of ethanolamine following intracellular hydrolysis of AEA in C6 cells over a similar concentration range as required for inhibition of AEA uptake (Fig. 4). Nefazodone also inhibited the uptake of [^3^H]PEA by HepG2 cells, with a pI_50_ value of 5.03±0.17 (IC_50_ value 9.3 µM), but, as with ketoconazole, inhibited the association of the ligand to wells over the same concentration range (data not shown).

### Ketoconazole and nefazodone inhibit FAAH activity

**Figure 5:**
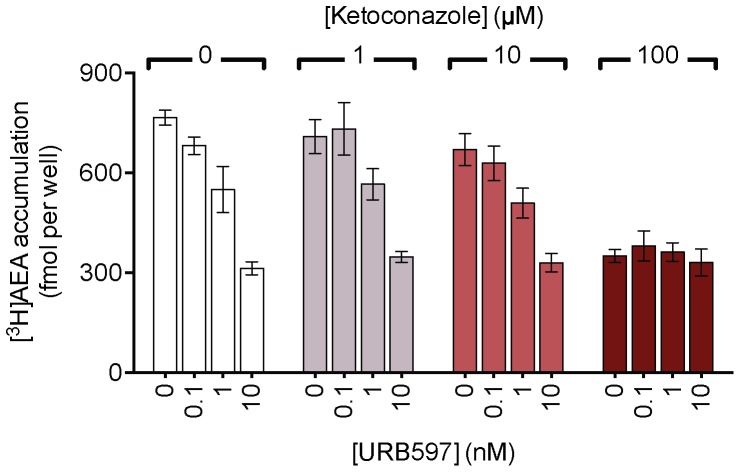
The effect of combinations of ketoconazole and URB597 upon the uptake of [^3^H]AEA into HepG2 cells. The cells were preincubated with the compounds for 10[^3^H]AEA (assay concentration 100 nM) and incubation for a further 4 min. Shown are means ± s.e.m., n = 3.

In many cell types using the experimental protocol used here, the uptake of AEA is driven by the activity of FAAH, since this removes the intracellularly accumulated AEA and hence preserves its gradient across the cell plasma membrane [32], [33]. This appears to be the case for the HepG2 and CaCo-2 cells, since URB597 produced a large reduction in the observed rate of AEA uptake in both cells (Fig. 1A,B, Fig. 3C). In the HepG2 cells, after a preincubation phase of 10 min with the test compounds and an incubation of 4 min with AEA, the inhibition produced by ketoconazole was not additive to that produced by URB597 (Fig. 5), suggesting that both compounds are acting along the same pathway. URB597 also reduced the uptake of PEA into the HepG2 cells, whereas the uptake of 2-AG was not affected by either this compound or by the MGL-selective inhibitor JZL184 [34] (Fig. 2B).

One explanation for the above findings is that ketoconazole (and nefazodone) affect AEA uptake in the cells due to a direct action upon FAAH. If this is the case, the compounds should be poor inhibitors of AEA uptake into cells lacking FAAH. Human PC-3 prostate cancer cells have a very low level of FAAH activity [35], [36] and are thus useful in this respect. Both compounds, as well as URB597, produced only modest inhibition of AEA uptake into PC-3 cells (Fig. 1C).

**Figure 6:**
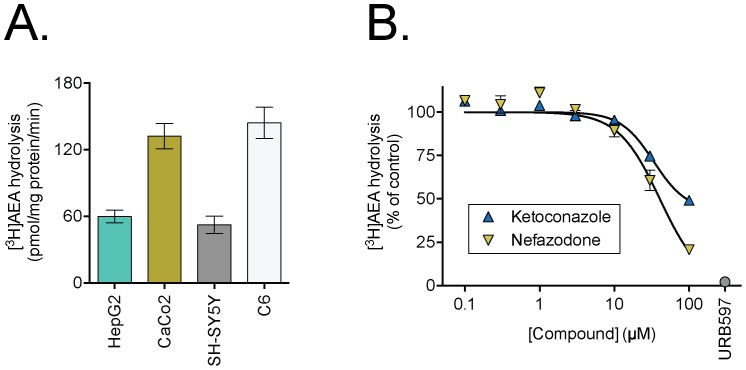
The effects of ketoconazole and nefazodone upon FAAH activity. Panel A. Hydrolysis of [^3^H]AEA by lysates of HepG2 and CaCo2 cells. Shown are means ± s.e.m., n = 3. Concurrent data for human SH-SY5Y neuroblastoma and rat C6 glioma cells are shown for comparison. Panel B. Inhibition of the hydrolysis of [^3^H]AEA in lysates of HepG2 cells by ketoconazole and nefazodone. Shown are means ± s.e.m. (unless enclosed by the symbols), n = 3.

In order to investigate the direct interaction of ketoconazole and nefazodone with FAAH, we measured FAAH activity in HepG2 and Caco2 cell lysates. Lysates from both HepG2 and Caco2 cell lines were able to hydrolyse AEA at rates not dissimilar to those seen with human SH-SY5Y neuroblastoma cells and rat C6 glioma cells (Fig. 6A). Using the HepG2 lysates, both ketoconazole and nefazodone were found to inhibit AEA hydrolysis (Fig. 6B). For nefazodone, the pI_50_ value was 4.39±0.04, corresponding to an IC_50_ value of 41 µM. For ketoconazole, the inhibition curve of best fit produced a maximum inhibition of 57±7%, with a pI_50_ value for the inhibitable component of 4.47±0.09 (IC_50_ value 34 µM). The lack of a complete inhibition of FAAH in the assay system used is a common phenomenon with lipophilic compounds in our hands (see e.g. [37]) and may represent a solubility issue, although it should be noted that a similar incomplete inhibition of AEA hydrolysis by intact C6 cells was found (Fig. 4B), whereas the inhibition of uptake was complete (Fig. 4A). The pI_50_ values for the inhibitable component of the FAAH activity seen in the lysates are reasonably in line with the pI_50_ values for the effects of the compounds upon AEA uptake in the intact cells.

## Discussion

In the present study we have demonstrated that ketoconazole inhibits the uptake of the endocannabinoid AEA into a variety of cell lines, and that this effect can be most simply explanined by the ability of the compound to inhibit FAAH. At first sight, the potency of the compound as an inhibitor of AEA uptake (IC_50_ value ∼20 µM for the FAAH-containing cells) is modest compared with the nanomolar affinity of the compound towards CYP51, CYP3A4 and CYP17 [17]–[19], and so might be considered a “pharmacological curiosity” without additional relevance. However, the potency of the compound towards AEA uptake is very similar to that seen for inhibition of 5-lipoxygenase, in cell-free preparations (28 µM) [20]. Thromboxane synthase is also inhibited *in vitro* by ketoconazole (IC_50_ value 40 µM [20], [38]). In intact rat peritoneal polymorphonucelar leukocytes, leukotriene B4 and 5-hydroxyeicosatetraenoic acid production from arachidonic acid was inhibited with IC_50_ values of 30 and 26 µM, respectively [20]. These authors demonstrated further than oral pretreatment with ketoconazole (10–40 mg/kg) inhibited in a dose-dependent manner ovalbumin-induced bronchoconstriction in sensitised guinea pigs, suggesting that leukotriene synthesis could be inhibited *in vivo* by the compound [20]. After oral administration of 400 mg of ketoconazole to volunteers, plasma levels of thromboxane B2 were unchanged with respect to placebo treatment. However, following ischaemia to the arm induced by a 10 min cuff, the increase in thromboxane B2 levels found after placebo was inhibited in the ketoconazole group [39]. In Sweden, ketoconazole is available as a shampoo (20 mg/ml) and until recently as tablets (200 mg; the dose could be doubled if deemed necessary), and in HIV-healthy volunteers, a C_max_ value of 5.3 µg/ml was found following 6 days of treatment with the 200 mg dose [40]. This corresponds to a plasma concentration of about 10 µM. Of course, this number does not take into account the considerable plasma protein binding of ketoconazole, but the uptake and FAAH experiments are also undertaken in the presence of serum albumin and so are comparable. Taken together, these data are consistent with the suggestion that ketoconazole affects AEA reuptake in pharmacologically relevant concentrations.

Ketoconazole can also affect PEA and 2-AG uptake at similar concentrations to those affecting AEA uptake. Whilst the effects upon PEA uptake are to be expected for a compound with FAAH-inhibitory properties [41] (see Fig. 2A), the effects upon 2-AG are more unexpected, since blockade of 2-AG hydrolysis does not reduce the intracellular accumulation of this endocannabinoid [42]–[44], a result also found here. However, AEA can inhibit 2-AG uptake [42], [44] suggesting communalities in their transport mechanisms, and so it is possible that an action of ketoconazole upon, for example, a fatty acid binding protein [7] may be involved in its effects upon 2-AG uptake, assuming, of course, that the effects are not simply non-specific, such as are seen in our experiments with the wells alone. An interaction with fatty acid binding proteins should be considered, given that 100 µM ketoconazole can compete with the arachidonoyl derivative 20-iodo-14,15-epoxyeicosa-8(Z)-enoyl-3-azidophenylsulfonamide for binding to a 47 kDa protein in cell membranes from U937 leukemic monocyte lymphoma cells [45]. PC-3 cells express FABP5 (E-FABP) and, to a lesser extent, FABP6 (IL-FABP), but not FABP7 (B-FABP) [46]. However an interaction between FABP5 and ketoconazole would have been expected to affect AEA uptake in these cells given that this fatty acid binding proteins acts as a carrier for the endocannabinoid [7]. An alternative possibility is that ketoconazole can interfere with the uptake of 2-AG by modulating processes downstream of the hydrolytic enzymes. This would be consistent with the finding that ketoconazole did not affect the rate of 2-AG hydrolysis in C6 glioma cells. Indeed, in astrocytoma cells, the uptake of 2-AG is reduced by the acyl-CoA synthetase inhibitor, triacsin C, suggesting that in these cells, the reincorporation of arachidonate into phospholipids rather than the hydrolysis of 2-AG is the driving force for the uptake of this endocannabinoid [42].

From an *in vitro* study to the situation *in vivo* is a large step to take, and further work is required to determine if the actions of ketoconazole upon AEA uptake and metabolism contribute to its pharmacological spectrum in whole animals, particularly following repeated administration. However, one area of research is of potential interest, namely that of cancer, given that modulation of the endocannabinoid system can affect cancer cell proliferation, migration and invasivity [5], [35], [47]–[51]. Ketoconazole has been used clinically in cancer for the treatment of chemotherapy-induced oral candidiasis [52]. In view of its ability to block hepatic CYP3A4, it may be useful as a way of reducing the doses needed of cytostatic agents such as docetaxel [53]. Ketoconazole has also been suggested as a potential treatment for prostate cancer, due to its ability to inhibit, albeit weakly, CYP17 involved in androgen biosynthesis [19], [54]. There is considerable interest in the design of designed multiple ligands, where effects upon multiple targets are optimised within a single molecule [55]. Within the endocannabinoid field, FAAH-TRPV1 and FAAH-cyclooxygenase inhibitors have been proposed as potential novel analgesics [56], [57]. The present study identifies ketoconazole as the first, to our knowledge, compound with inhibitory effects towards both FAAH and CYP17. It may be possible to utilise ketoconazole as a template for the design of dual-action FAAH-CYP17 inhibitors lacking the liver toxicity of the parent compound, as a novel treatment strategy for the treatment of prostate cancer.

## References

[1] Roques BP, Fournié-Zaluski MC, Wurm M (2012) Inhibiting the breakdown of endogenous opioids and cannabinoids to alleviate pain. Nat Rev Drug Discov 11: 292–310. 22460123 10.1038/nrd3673

[2] Di Marzo V, Ligresti A, Cristino L (2009) The endocannabinoid system as a link between homoeostatic and hedonic pathways involved in energy balance regulation. Int J Obes (Lond) 33 Suppl 2S18–24. 19528974 10.1038/ijo.2009.67

[3] Battista N, Meccariello R, Cobellis G, Fasano S, Di Tommaso M, et al (2012) The role of endocannabinoids in gonadal function and fertility along the evolutionary axis. Mol Cell Endocrinol 355: 1–14. 22305972 10.1016/j.mce.2012.01.014

[4] Idris AI, Ralston SH (2012) Role of cannabinoids in the regulation of bone remodeling. Front Endocrinol (Lausanne) 3: 136. 23181053 10.3389/fendo.2012.00136 PMC3499879

[5] Pisanti S, Picardi P, D'Alessandro A, Laezza C, Bifulco M (2013) The endocannabinoid signaling system in cancer. Trends Pharmacol Sci 34: 273–282. 23602129 10.1016/j.tips.2013.03.003

[6] Fowler CJ (2013) Transport of endocannabinoids across the plasma membrane and within the cell. FEBS J 280: 1895–1904. 23441874 10.1111/febs.12212

[7] Kaczocha M, Glaser S, Deutsch D (2009) Identification of intracellular carriers for the endocannabinoid anandamide. Proc Natl Acad Sci USA 106: 6375–6380. 19307565 10.1073/pnas.0901515106 PMC2669397

[8] Oddi S, Fezza F, Pasquariello N, D'Agostino A, Catanzaro G, et al (2009) Molecular identification of albumin and Hsp70 as cytosolic anandamide-binding proteins. Chem Biol 16: 624–632. 19481477 10.1016/j.chembiol.2009.05.004

[9] Fu J, Bottegoni G, Sasso O, Bertorelli R, Rocchia W, et al (2011) A catalytically silent FAAH-1 variant drives anandamide transport in neurons. Nat Neurosci 15: 64–69. 22101642 10.1038/nn.2986 PMC3245783

[10] Leung K, Elmes MW, Glaser ST, Deutsch DG, Kaczocha M (2013) Role of FAAH-like anandamide transporter in anandamide inactivation. PLoS One 8: e79355. 24223930 10.1371/journal.pone.0079355 PMC3817039

[11] Deutsch DG, Chin SA (1993) Enzymatic synthesis and degradation of anandamide, a cannabinoid receptor agonist. Biochem Pharmacol 46: 791–796. 8373432 10.1016/0006-2952(93)90486-g

[12] Ueda N, Tsuboi K, Uyama T (2013) Metabolism of endocannabinoids and related *N*-acylethanolamines: Canonical and alternative pathways. FEBS J 280: 1874–1894. 23425575 10.1111/febs.12152

[13] Hermanson DJ, Hartley ND, Gamble-George J, Brown N, Shonesy BC, et al (2013) Substrate-selective COX-2 inhibition decreases anxiety via endocannabinoid activation. Nat Neurosci 16: 1291–1298. 23912944 10.1038/nn.3480 PMC3788575

[14] Gatta L, Piscitelli F, Giordano C, Boccella S, Lichtman A, et al (2012) Discovery of prostamide F_2α_ and its role in inflammatory pain and dorsal horn nociceptive neuron hyperexcitability. PLoS ONE 7: e31111. 22363560 10.1371/journal.pone.0031111 PMC3283613

[15] Snider NT, Walker VJ, Hollenberg PF (2010) Oxidation of the endogenous cannabinoid arachidonoyl ethanolamide by the cytochrome p450 monooxygenases: physiological and pharmacological implications. Pharmacol Rev 62: 136–154. 20133390 10.1124/pr.109.001081 PMC2835397

[16] Snider NT, Nast JA, Tesmer LA, Hollenberg PF (2009) A cytochrome P450-derived epoxygenated metabolite of anandamide is a potent cannabinoid receptor 2-selective agonist. Mol Pharmacol 75: 965–972. 19171674 10.1124/mol.108.053439 PMC2684935

[17] Warrilow AG, Parker JE, Kelly DE, Kelly SL (2013) Azole affinity of sterol 14α-demethylase (CYP51) enzymes from *Candida albicans* and *Homo sapiens* . Antimicrob Agents Chemother 57: 1352–1360. 23274672 10.1128/AAC.02067-12 PMC3591892

[18] Sai Y, Dai R, Yang TJ, Krausz KW, Gonzalez FJ, et al (2000) Assessment of specificity of eight chemical inhibitors using cDNA-expressed cytochromes P450. Xenobiotica 30: 327–343. 10821163 10.1080/004982500237541

[19] Potter GA, Barrie SE, Jarman M, Rowlands MG (1995) Novel steroidal inhibitors of human cytochrome P450_17α_ (17α-hydroxylase-C_17,20_-lyase): potential agents for the treatment of prostatic cancer. J Med Chem 38: 2463–2471. 7608911 10.1021/jm00013a022

[20] Beetens JR, Loots W, Somers Y, Coene MC, De Clerck F (1986) Ketoconazole inhibits the biosynthesis of leukotrienes *in vitro* and *in vivo* . Biochem Pharmacol 35: 883–891. 3006695 10.1016/0006-2952(86)90072-9

[21] Rakhshan F, Day T, Blakeley R, Barker E (2000) Carrier-mediated uptake of the endogenous cannabinoid anandamide in RBL-2H3 cells. J Pharmacol Exp Ther 292: 960–967. 10688610

[22] Sandberg A, Fowler CJ (2005) Measurement of saturable and non-saturable components of anandamide uptake into P19 embryonic carcinoma cells in the presence of fatty acid-free bovine serum albumin. Chem Phys Lipids 134: 131–139. 15784231 10.1016/j.chemphyslip.2004.12.010

[23] Bradford MM (1976) A rapid and sensitive method for the quantitation of microgram quantities of protein utilizing the principle of protein-dye binding. Anal Biochem 72: 248–254. 942051 10.1016/0003-2697(76)90527-3

[24] Boldrup L, Wilson SJ, Barbier AJ, Fowler CJ (2004) A simple stopped assay for fatty acid amide hydrolase avoiding the use of a chloroform extraction phase. J Biochem Biophys Methods 60: 171–177. 15262451 10.1016/j.jbbm.2004.04.020

[25] Beltramo M, Stella N, Calignano A, Lin SY, Makriyannis A, et al (1997) Functional role of high-affinity anandamide transport, as revealed by selective inhibition. Science 277: 1094–1097. 9262477 10.1126/science.277.5329.1094

[26] Ortar G, Ligresti A, De Petrocellis L, Morera E, Di Marzo V (2003) Novel selective and metabolically stable inhibitors of anandamide cellular uptake. Biochem Pharmacol 65: 1473–1481. 12732359 10.1016/s0006-2952(03)00109-6

[27] Karlsson M, Påhlsson C, Fowler CJ (2004) Reversible, temperature-dependent, and AM404-inhibitable adsorption of anandamide to cell culture wells as a confounding factor in release experiments. Eur J Pharm Sci 22: 181–189. 15158903 10.1016/j.ejps.2004.03.009

[28] Fowler CJ, Tiger G, Ligresti A, López-Rodríguez ML, Di Marzo V (2004) Selective inhibition of anandamide cellular uptake versus enzymatic hydrolysis - a difficult issue to handle. Eur J Pharmacol 492: 1–11. 15145699 10.1016/j.ejphar.2004.03.048

[29] Ortega-Gutiérrez S, Hawkins E, Viso A, López-Rodríguez M, Cravatt B (2004) Comparison of anandamide transport in FAAH wild-type and knockout neurons: evidence for contributions by both FAAH and the CB1 receptor to anandamide uptake. Biochemistry 43: 8184–8190. 15209515 10.1021/bi049395f

[30] Thors L, Fowler CJ (2006) Is there a temperature-dependent uptake of anandamide into cells? Br J Pharmacol 149: 73–81. 16865094 10.1038/sj.bjp.0706831 PMC1629410

[31] Kathuria S, Gaetani S, Fegley D, Valiño F, Duranti A, et al (2003) Modulation of anxiety through blockade of anandamide hydrolysis. Nat Med 9: 76–81. 12461523 10.1038/nm803

[32] Day T, Rakhshan F, Deutsch D, Barker E (2001) Role of fatty acid amide hydrolase in the transport of the endogenous canabinoid anandamide. Mol Pharmacol 59: 1369–1375. 11353795 10.1124/mol.59.6.1369

[33] Deutsch D, Glaser S, Howell J, Kunz J, Puffenbarger R, et al (2001) The cellular uptake of anandamide is coupled to its breakdown by fatty-acid amide hydrolase. J Biol Chem 276: 6967–6973. 11118429 10.1074/jbc.M003161200

[34] Long JZ, Li W, Booker L, Burston JJ, Kinsey SG, et al (2009) Selective blockade of 2-arachidonoylglycerol hydrolysis produces cannabinoid behavioral effects. Nat Chem Biol 5: 37–44. 19029917 10.1038/nchembio.129 PMC2605181

[35] Endsley M, Thill R, Choudhry I, Williams C, Kajdacsy-Balla A, et al (2008) Expression and function of fatty acid amide hydrolase in prostate cancer. Int J Cancer 123: 1318–1326. 18566995 10.1002/ijc.23674 PMC2548421

[36] Thors L, Eriksson J, Fowler CJ (2007) Inhibition of the cellular uptake of anandamide by genistein and its analogue daidzein in cells with different levels of fatty acid amide hydrolase-driven uptake. Br J Pharmacol 152: 744–750. 17676056 10.1038/sj.bjp.0707401 PMC2190009

[37] Vandevoorde S, Jonsson K-O, Fowler CJ, Lambert DM (2003) Modifications of the ethanolamine head in *N*-palmitoylethanolamine: synthesis and evaluation of new agents interfering with the metabolism of anandamide. J Med Chem 46: 1440–1448. 12672243 10.1021/jm0209679

[38] Tolman EL, Fuller BL (1983) Inhibition of thromboxane synthesis in guinea pig lung and human platelets by clotrimazole and other imidazole antifungals. Biochem Pharmacol 32: 3488–3490. 6316985 10.1016/0006-2952(83)90383-0

[39] Lelcuk S, Huval WV, Valeri CR, Shepro D, Hechtman HB (1984) Inhibition of ischemia-induced thromboxane synthesis in man. J Trauma 24: 393–396. 6325720 10.1097/00005373-198405000-00004

[40] Sekar VJ, Lefebvre E, De Pauw M, Vangeneugden T, Hoetelmans RM (2008) Pharmacokinetics of darunavir/ritonavir and ketoconazole following co-administration in HIV-healthy volunteers. Br J Clin Pharmacol 66: 215–221. 18460033 10.1111/j.1365-2125.2008.03191.x PMC2492919

[41] Hillard C, Jarrahian A (2005) Accumulation of anandamide: evidence for cellular diversity. Neuropharmacology 48: 1072–1078. 15910883 10.1016/j.neuropharm.2004.12.012

[42] Beltramo M, Piomelli D (2000) Carrier-mediated transport and enzymatic hydrolysis of the endogenous cannabinoid 2-arachidonylglycerol. NeuroReport 11: 1231–1235. 10817598 10.1097/00001756-200004270-00018

[43] Fowler CJ, Ghafouri N (2008) Does the hydrolysis of 2-arachidonoylglycerol regulate its cellular uptake? Pharmacol Res 58: 72–76. 18675915 10.1016/j.phrs.2008.07.002

[44] Chicca A, Marazzi J, Nicolussi S, Gertsch J (2012) Evidence for bidirectional endocannabinoid transport across cell membranes. J Biol Chem 287: 34660–34682. 22879589 10.1074/jbc.M112.373241 PMC3464571

[45] Chen Y, Falck JR, Manthati VL, Jat JL, Campbell WB (2011) 20-Iodo-14,15-epoxyeicosa-8(Z)-enoyl-3-azidophenylsulfonamide: photoaffinity labeling of a 14,15-epoxyeicosatrienoic acid receptor. Biochemistry 50: 3840–3848. 21469660 10.1021/bi102070w PMC3100183

[46] Tölle A, Suhail S, Jung M, Jung K, Stephan C (2011) Fatty acid binding proteins (FABPs) in prostate, bladder and kidney cancer cell lines and the use of IL-FABP as survival predictor in patients with renal cell carcinoma. BMC Cancer 11: 302. 21767383 10.1186/1471-2407-11-302 PMC3199863

[47] Freimuth N, Ramer R, Hinz B (2010) Antitumorigenic effects of cannabinoids beyond apoptosis. J Pharmacol Exp Ther 332: 336–344. 19889794 10.1124/jpet.109.157735

[48] Fowler CJ, Gustafsson SB, Chung SC, Persson E, Jacobsson SOP, et al (2010) Targeting the endocannabinoid system for the treatment of cancer - a practical view. Curr Top Med Chem 10: 814–827. 20370711 10.2174/156802610791164201

[49] Díaz-Laviada I (2011) The endocannabinoid system in prostate cancer. Nat Rev Urol 8: 553–561. 21912423 10.1038/nrurol.2011.130

[50] Guindon J, Hohmann AG (2011) The endocannabinoid system and cancer: therapeutic implication. Br J Pharmacol 163: 1447–1463. 21410463 10.1111/j.1476-5381.2011.01327.x PMC3165955

[51] Velasco G, Sanchez C, Guzmán M (2012) Towards the use of cannabinoids as antitumour agents. Nat Rev Cancer 12: 436–444. 22555283 10.1038/nrc3247

[52] Worthington HV, Clarkson JE, Khalid T, Meyer S, McCabe M (2010) Interventions for treating oral candidiasis for patients with cancer receiving treatment. Cochrane Database Syst Rev 2010: CD001972. 10.1002/14651858.CD001972.pub4 PMC7063978 20614427

[53] Zee YK, Goh BC, Lee SC (2012) Pharmacologic modulation strategies to reduce dose requirements of anticancer therapy while preserving clinical efficacy. Future Oncol 8: 731–749. 22764771 10.2217/fon.12.53

[54] Vasaitis TS, Bruno RD, Njar VC (2011) CYP17 inhibitors for prostate cancer therapy. J Steroid Biochem Mol Biol 125: 23–31. 21092758 10.1016/j.jsbmb.2010.11.005 PMC3047603

[55] Morphy R, Rankovic Z (2005) Designed multiple ligands. An emerging drug discovery paradigm. J Med Chem 48: 6523–6543. 16220969 10.1021/jm058225d

[56] Starowicz K, Di Marzo V (2013) Non-psychotropic analgesic drugs from the endocannabinoid system: “magic bullet” or “multiple-target” strategies? Eur J Pharmacol 716: 41–53. 23500197 10.1016/j.ejphar.2013.01.075

[57] Fowler CJ, Naidu PS, Lichtman A, Onnis V (2009) The case for the development of novel analgesic agents targeting both fatty acid amide hydrolase and either cyclooxygenase or TRPV1. Br J Pharmacol 156: 412–419. 19226258 10.1111/j.1476-5381.2008.00029.x PMC2697682

